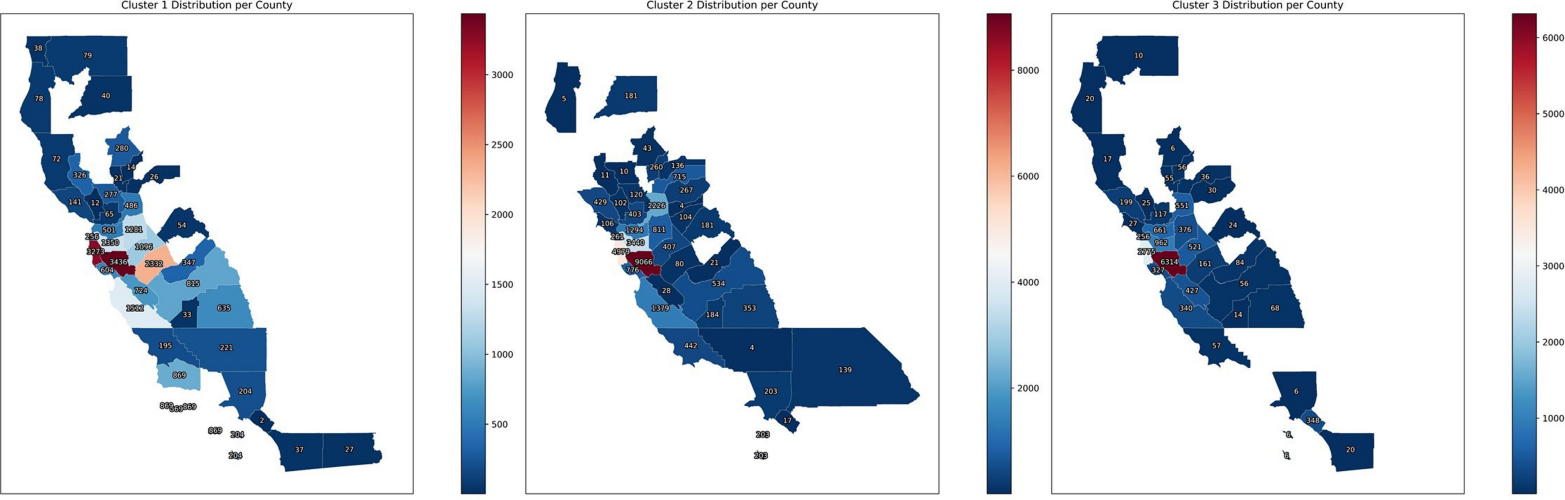# A Machine Learning Exploration of Social Determinants of Health and Hospital-Onset Bacteremia, Northern California, 2019–2023

**DOI:** 10.1017/ash.2024.300

**Published:** 2024-09-16

**Authors:** Guillermo Rodriguez Nava, Eugenia Miranti, Karen McIntyre, Sulwan AlGain, John Shepard, Jorge Salinas, Mindy Sampson

**Affiliations:** Stanford University School of Medicine; Stanford Healthcare; Stanford University; King Faisal Specialist Hospital and Research Center

## Abstract

**Introduction:** Social determinants of health can impact healthcare-associated infections. Hospital-onset bacteremia (HOB) may lead to poor outcomes, increased length of stay, and increased cost of care. We explored the association of social determinants of health and HOB. **Methods:** We retrieved blood culture data at Stanford Health Care from May 2019 to October 2023. We identified blood cultures ordered ≥4 days of admission. To evaluate the association between social determinants of health and HOB, we employed an unsupervised machine learning approach (K-Means clustering) to discern patterns in HOB rates based on the Social Vulnerability Index (SVI). The SVI indicates the relative vulnerability of every U.S. Census tract. It ranks the tracts on 16 measures of vulnerability across 4 themes: socioeconomic factors, household characteristics, racial and ethnic minority status, and housing/transportation aspects. The number of clusters was determined using the Elbow Method (Figure 1). **Results:** Out of 209,947 blood cultures from 23,938 unique patients with a California address, we identified 81,653 blood cultures collected after 4 days (40%). The K-Means clustering algorithm identified 3 distinct clusters within the Californian census tracts, suggesting heterogeneity in the relationship between SVI and HOB (Figure 2). Cluster 1 had a higher SVI (median 0.73, range 0.46 – 0.99), with logistic regression indicating a positive SVI-HOB association (OR 4.84, 95% CI 4.02 – 4.81, p <.001). Cluster 2, had a median SVI of 0.29 (range 0.0009 – 0.78), also showed a positive association between SVI and HOB (OR 1.67, 95% CI 1.4 – 1.89, p <.001), aligning with trends of higher infection risks in more vulnerable groups. In contrast, Cluster 3 had a median SVI of 0.22 (range 0.002 –0.84). In this cluster, the SVI showed a negative association with HOB (OR 0.24, 95% CI 0.18 – 0.31, p <.001). Cluster 3 was the cluster with the least number of subjects (15,000, versus 21,761 for Cluster 1 and 29,762 for Cluster 2). Most subjects in Cluster 3 resided in Santa Clara County, whereas those in Clusters 1 and 2 were spread across Santa Clara, San Mateo, Alameda, Merced, and Sacramento Counties (Figure 3). **Conclusions:** Advanced techniques can be used to explore the complex interplay between social determinants of health and healthcare-associated infections and could guide the development of community-specific strategies to improve outcomes.

**Figure:**
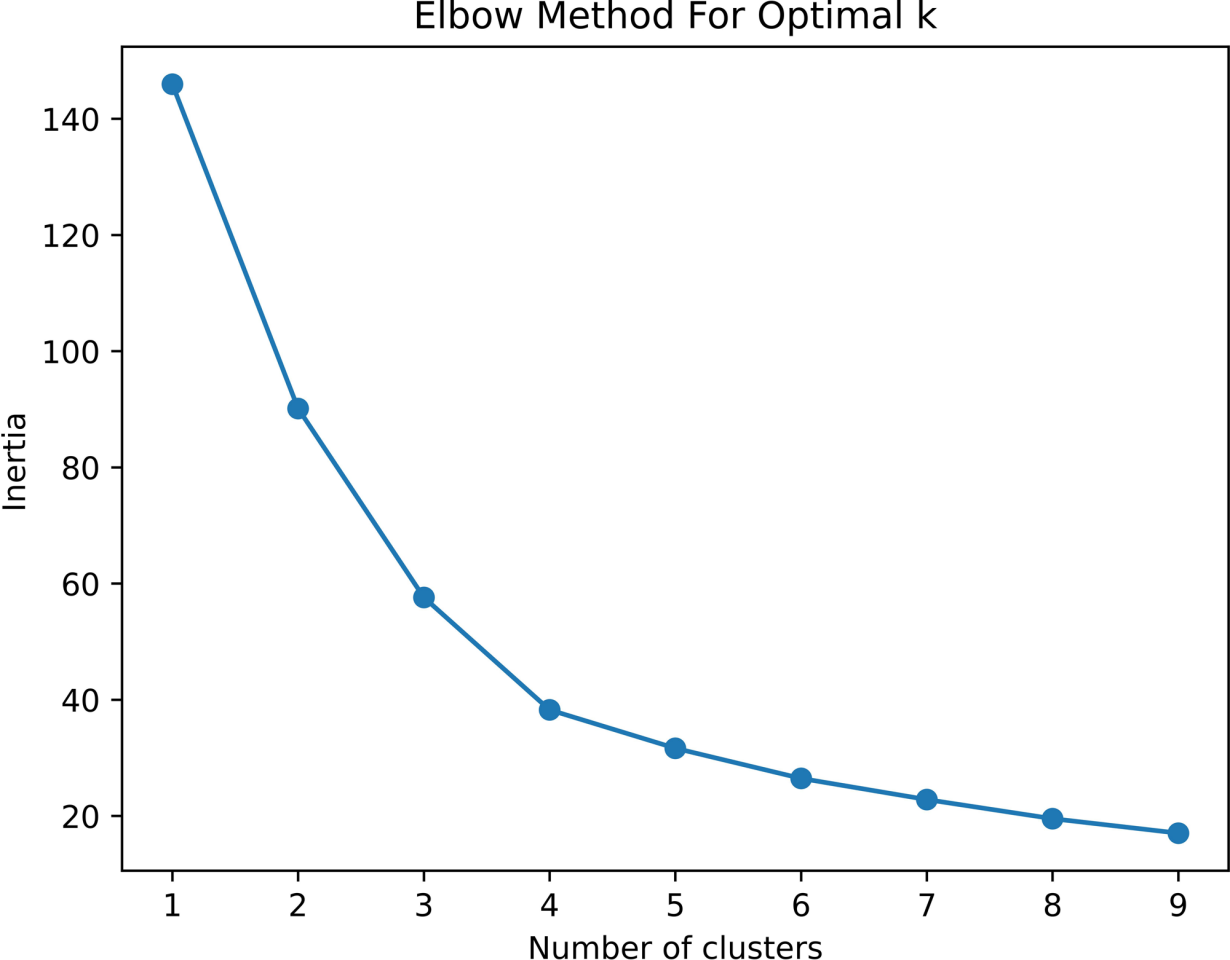

**Figure:**
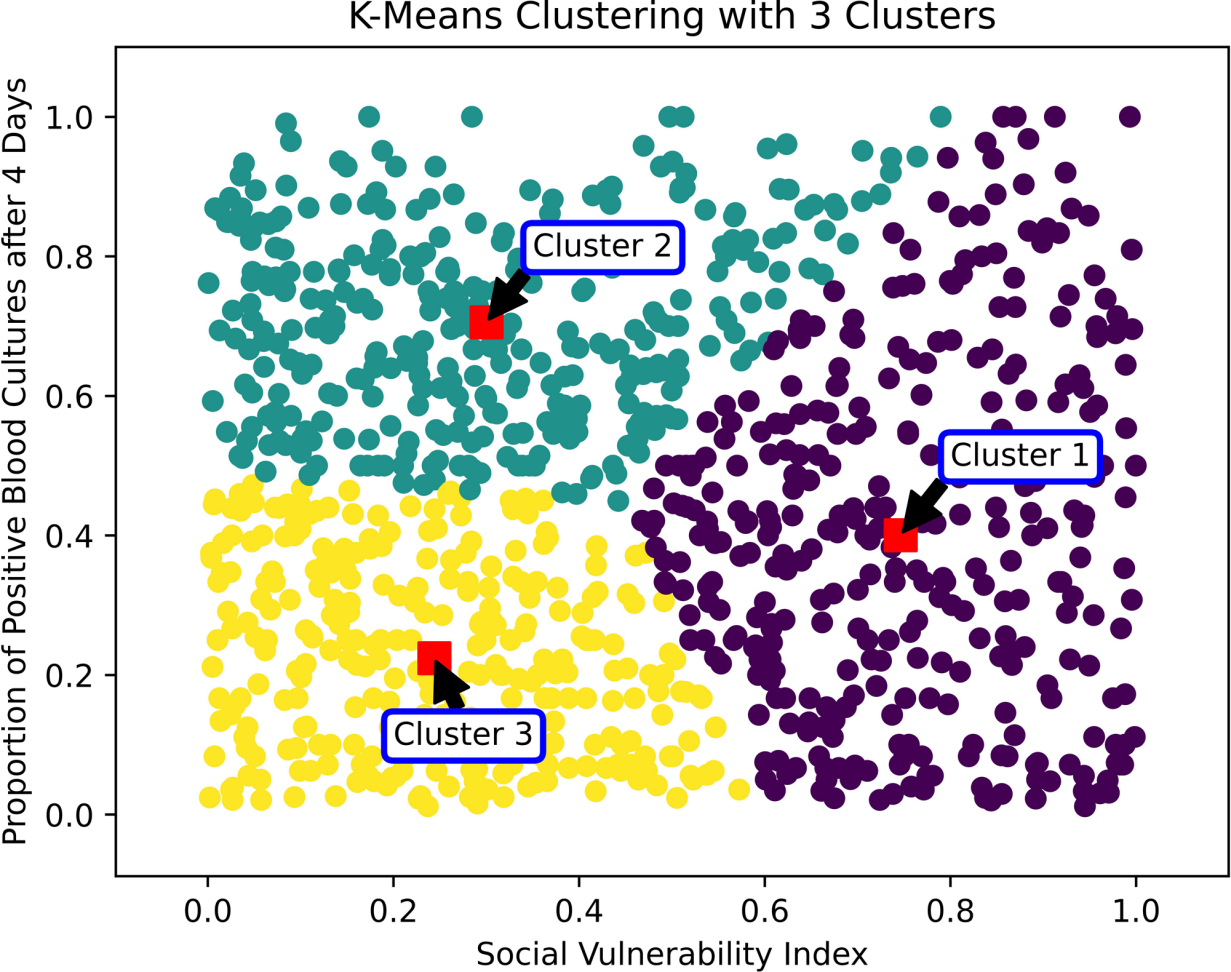

**Figure:**